# A cross sectional study of upper extremity strength ten days after a stroke; relationship between patient-reported and objective measures

**DOI:** 10.1186/s12883-015-0436-8

**Published:** 2015-10-01

**Authors:** Hanna C. Persson, Anna Danielsson, Katharina S. Sunnerhagen

**Affiliations:** Department of Clinical Neuroscience and Rehabilitation, Institute of Neuroscience and Physiology, Sahlgrenska Academy, University of Gothenburg, Gothenburg, Sweden; Centre for Person-Centred Care, (GPCC) Sahlgrenska Academy, University of Gothenburg, Gothenburg, Sweden; Unit of Physiotherapy, Division of Health and Rehabilitation, Institute of Neuroscience and Physiology, Sahlgrenska Academy, University of Gothenburg, Gothenburg, Sweden

**Keywords:** Stroke, Upper extremity, Muscle strength, Self report, Outcome measure

## Abstract

**Background:**

Reduced upper extremity function early after a stroke is common, and a combination of strength capacity and patient-reported measures contribute to setting realistic goals. The validity of the patient’s perception of upper extremity strength in relation to objective strength assessments early after a stroke needs to be clarified. The objective was to investigate the relationship between perceived upper extremity strength and measured hand strength at ten days post-stroke.

**Methods:**

This study of 99 patients with reduced upper extremity function at 3 days post stroke, were consecutively included from a stroke unit to the Stroke Arm Longitudinal Study at the University of Gothenburg, (the SALGOT-study). The correlations between two questions from the Stroke Impact Scale (SIS 1a and 1b), and a dynamometer measure of hand strength values (percentage of normative values) were investigated. In order to explain differences between the two types of measurements, the accordance between perceived strength in a dichotomized SIS and objective measures was explored. In SIS 1a and 1b, 1–3 points correspond to reduced strength (<80 % or normative strength values). In SIS 1a and 1b, 4–5 points correspond to normal strength (≥80 % of normative strength values).

**Results:**

The correlation between the measured strength values and perceived arm strength was rho 0.82 (*p* = <0.001) and with perceived grip strength rho 0.87 (*p* = <0.001). Using the dichotomized SIS and the 80 % cut-off correctly classified arm strength in 81 % and grip strength in 84 % of the patients, with a sensitivity of 0.86-0.87, a specificity of 0.62-0.77, positive predicted values of 0.87-0.91 and negative predicated values of 0.64-0.67.

**Discussion:**

The discrepancy between assessed strength capacity and self-perceived strength highlights the importanceof including self-perceived assessments early after stroke, in order to increase knowledge of a patient'sawareness of functioning or lack thereof.

**Conclusions:**

Ten days after stroke in patients without severe cognitive disabilities, this study suggests that despite high correlations between measures, an objective assessment of arm and hand strength does not always reflect the patient’s perspective. A combination of self-reported and objective strength assessment is requested to enhance in setting of realistic goals early after stroke.

**Trial registration:**

ClinicalTrials.gov: NCT01115348, May 3, 2010

## Background

Care and rehabilitation strive to become more person-centered [1, 2]. In accordance with this concept, a patient’s experiences and knowledge are important assets to achieve care of high quality. Self-reported measurements offer unique information [3, 4], could give insight into a patients deficits in a practical way [3], and covers information that is not obtained with objective outcome measures and vice versa [4]. Self-reported measurements are rarely studied after a stroke compared to in other diseases [3, 5]. A discrepancy between actual strength capacity and patient-reported capacity has previously been shown [3, 4] and improved understanding of the relationship between the two assessments is important to capture the different perspectives [3].

Rehabilitation after stroke should take place at a stroke unit [6] and start within the first days after onset [7]. Recovery of function predominantly occurs within the first few weeks [8, 9]. Function in the upper-extremity can be investigated with objective measurements of grip strength which have been shown to be sensitive and useful [10, 11] and approximately 80 % of normative strength values correspond to normal (maximal) functional performance [12]. Another measure of strength is the patients’ own view of his/her strength, but its relation to clinical assessments needs to be clarified. A combination of objective measured and patient reported function may enhance the setting of realistic goals [2] which is of importance for rehabilitation efficiency as well as reducing the in-patient time in hospital. A patient’s lack of awareness of their impaired function complicates the rehabilitation process and may lead to poorer outcomes [13]. To our knowledge, there have been no studies investigating patients’ perceptions of strength in their upper extremity in the early days post stroke. The purpose of this cross sectional study was to investigate the relationship between perceived upper extremity strength and clinically measured hand strength at ten days post-stroke.

## Methods

### Design and participants

Over a period of 18 months (2009–2010), 117 patients were consecutively enrolled from the largest of three stroke units at the Sahlgrenska University Hospital in Gothenburg, Sweden, to the Stroke Arm Longitudinal Study at the University of Gothenburg, (the SALGOT-study) [14]. This study is a primary report from the SALGOT study with the following inclusion criteria: 1) first clinical stroke (ischemic or hemorrhagic) diagnosed based on the criteria by the World Health Organization; 2) impaired upper extremity on day three after onset, defined as <57 points on the Action Research Arm Test (ARAT) (0–57 points) [15–17]; 3) received treatment in the stroke unit within three days (±1); 4) resident in the Gothenburg urban area; 5) ≥18 years of age. The exclusion criteria were as follows: 1) an upper-extremity injury/condition prior to the stroke, which limited the functional use of the affected arm and/or hand; 2) severe multi-impairment or diminished physical condition before the stroke that will affect the arm function; 3) short life expectancy, less than 12 months due to other illness or severity of stroke injury; 4) non-Swedish speaking. In addition, incomplete answers in the strength domain (domain one) of the Stroke Impact Scale (SIS) [18, 19] or incomplete objective measure of hand strength [20] ten days after stroke onset was used as the 5^th^ exclusion criterion. This resulted in exclusion of 18 patients from the SALGOT-population, due to severe communication disorder (*n* = 13), cognitive deficits (*n* = 2), fatigue (*n* = 2) or incomplete objective strength assessment (*n* = 1). Finally, this inclusion process resulted in 99 patients. The SALGOT study received ethical approval by the Regional Ethical Review Board in Gothenburg. All patients or next of kin gave informed written consent for participation. The study is registered at the clinical trails.gov: NCT01115348.

### Measurements and procedures

Two main measures were used on day ten post-stroke and administered in the following order by a physiotherapist. First, the strength in the paretic hand was measured in Newton (N) with a dynamometer (the JAMAR Hand Dynamometer, Sammons Preston, Chicago) [20]. To increase the ability to participate even if a patient had low muscle strength, patients rested their arm and hand on a table during the measurement. Verbal encouragement from the physiotherapist during the test situation was given. The average of three trials was used and reported as the percentage of normative values, adjusted for age and for dominant/non dominant hand [21]. A cut off of 80 % of the normative strength values [21] was chosen to delineate normal strength versus reduced strength. In the subsequent text ≥80 % corresponds to normal strength, and <80 % as reduced strength. Following this test the patient’s perceived strength was assessed with the SIS version 3.0 Swedish version [18, 19], which covers different perspectives after a stroke, including arm and hand strength. The strength domain includes two questions regarding arm and hand strength during the last week. In this study, the following two questions from the strength domain (domain 1), were used: *“In the past week, how would you rate the strength of your; 1a) Arm that was most affected by your stroke? 1b) Grip of your hand that was most affected by your stroke?”* The patient rated his/her strength on a verbal, five point ordinal scale where 1 corresponds to *no strength at all*, 2 to *a little strength*, 3 to *some strength*, 4 to *quite a bit of strength* and 5 to *a lot of strength*. In this study a dichotomization of the ordinal scale was done with 1–3 corresponding to “perceived reduced strength in arm/hand” and 4–5 corresponding to “perceived good strength in the arm/hand”.

The patient’s neurological deficit was described using the arrival score performed at the hospital on the National Institute of Health Stroke Scale (NIHSS) [22]. As a screening of cognitive function, the COG4 consisting of following items from NIHSS; orientation, executive function, language and inattention (0–9 points, where 0 indicates no cognitive reduction) were derived [23]. On ten days post-stroke, the Fugl-Meyer Assessment for Upper Extremity (FMA-UE) [24, 25] 0–66 points, was applied to evaluate the upper extremity motor function and to evaluate sensation (0–12 points). The patient’s level of cooperation, aletness and language skills was screened using the pre-screening of the Barrow Neurological Institute Screen for Higher Cerebral Function (BNIS, 1–9 points, where 9 indicates ability to participate in an assessment) [26]. Clinical characteristics were gathered from the patient’s chart and the Swedish National Stroke Register [27].

### Statistics

The statistical analyses were conducted using the IBM Statistical Package for Social Sciences (SPSS) version 21.0, with a statistically significant level set to *p* < 0.05. Spearman’s rank correlation test (rho) was used to explore correlations between SIS 1a, 1b, and the measured strength (percentage of normative values in N).

To further investigate the accordance between perceived arm and hand strength and objectively measured strength, the dichotomization of the strength values was used. Reduced objective strength was estimated to be equal to the perceived reduced strength in the arm/hand, and normal objective strength was used to correspond to perceived good strength in the arm/hand. Using 2-way contingency tables, the percentage of correctly classified patients, the sensitivity, specificity, negative predicted values (NPV) and the positive predicted values (PPV) including a 95 % exact confidence interval (CI) [28] were calculated.

## Results

**Fig. 1:**
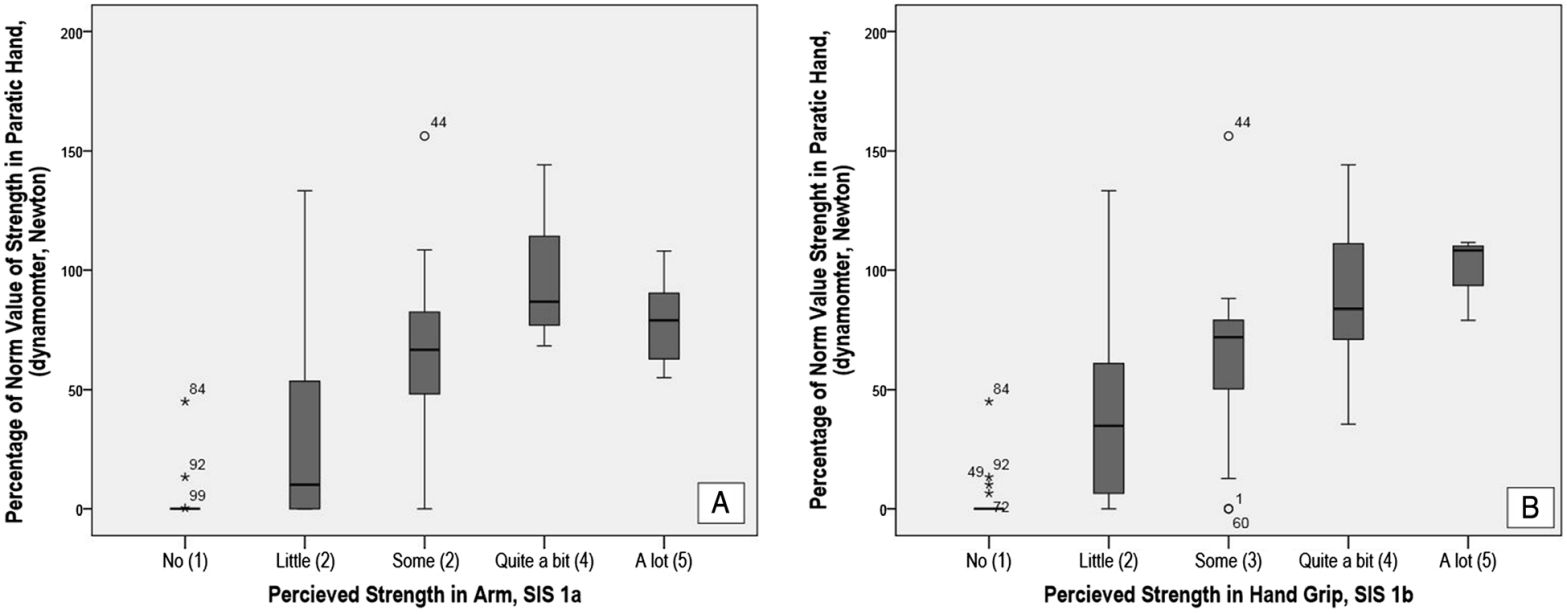
Objective strength at different levels of perceived strength in the paretic arm and hand. **a** illustrates objective strength (percentages of normative dynamometer strength values) in relation to self-reported arm strength. **b** illustrates the objective strength in the relation to self-reported hand strength. Abbreviations: *Dynamometer*; JAMAR Hand Dynamometer; *SIS*, Stroke Impact Scale questions 1A and 1B

Demographical data are given in Table 1. Fifty-seven percent were men, and the mean age at stroke onset was 67.4 years. The average of normative strength values at ten days after stroke, assessed with dynamometer, was less than 50 %. Few patients had reduced ability to participate in the test situation or had cognitive defects assessed with screening tests. Figure 1 illustrates the different levels of perceived strength (SIS) in relation to the objective strength measure expressed as a percentage of normative values ten days post-stroke. As shown, perceived strength (SIS) for arm and hand grip were similar (Fig. 1a and b). The widest disparities were found in the categories *a little strength* (2 points) and *some strength* (3 p). Please note that the category *a lot of strength* (5 points) was rated by only 5 (SIS 1a) and 4 (SIS 1b) patients, respectively. The correlations between the measured (percentage of normative strength values) and perceived arm (SIS 1a) and hand grip (SIS 1b) strength were rho 0.82 (*p* = <0.001) and rho 0.87 (*p* = <0.001), respectively.

**Table 1:** Characteristics of the study population (*n* 99)

Characteristics	
Female/Male, n	42/57
Age, years mean (SD)	67.4 (12.7)
Stroke/intervention	
Ischemic stroke/intracerebral hemorrhage, *n*	81/18
Hemisphere of stroke, n	
Right	54
Left	39
Cerebellum	1
Brain steam	1
Bilateral	4
Thrombolysis/Thrombectomy, n	11/4
At arrival to hospital	
NIHSS*, median (Q1-Q3)	6.0 (3–11)
Arm-score*, median (Q1-Q3)	2 (1–4)
COG4*, median (Q1-Q3)	0 (0–1)
At day 10	
Upper extremity function, FMA-UE*, median (Q1-Q3), n = 83	48 (6–66)
Sensation FMA-UE*, median (Q1,-Q3), n = 83	11 (2–12)
Paretic hand strength, percentage of normative values, mean (SD)	46.1 (44.5)
Cognitive function, Pre-BNIS*, median (Q1-Q3)	9 (9–9)
At hospital/at home, n	88/11
Training with physiotherapist or occupational therapist, n	
≥ 3 times per week, hospital based rehabilitation	88
Once time per week, outpatient rehabilitation	1
No rehabilitation	10

*Range of each measurement, best score indicated in bold: Pre-BNIS 0–**9**; COG4 **0**–9; FMA-UE 0–**66**; FMA-UE sensation 0–**12**; NIHSS **0**–42; NIHSS Arm score **0**–8

Abbreviations: *Pre-BNIS*; pre-screening of Barrow Neurological Institute Screen for Higher Cerebral Function; *FMA-UE*, The Fugl-Meyer Assessment for Upper Extremity; *NIHSS*, National Institutes of Health Stroke Scale, COG4 includes level of consciousness questions and commands, best language, extinction and inattention; *SD*, standard deviation; *Q1-Q3* 1^st^ and 3^th^ quartiles

Table 2 shows the accordance of dichotomized perceived strength and objectively measured strength using the 80 % cut-off. In SIS 1a, 9 patients rated perceived *good* strength in the arm while the objective measure showed *reduced* strength. Similar numbers were shown in SIS 1b (*n* = 10). Additionally, in SIS 1a, 10 patients rated *reduced* strength in arm while the objective measure showed *good* strength. Similar numbers were found in SIS hand 1b (*n* = 6). Correctly classified patients using the 80 % cut-off were 81 % (CI 95 % 0.717-0.880) in arm strength and 84 % (CI 95 % 0.751-0.905) in grip strength. The PPV in SIS arm (1a) and SIS hand (1b) were at 0.87-0.91, and the NPV were at 0.64-0.67.

**Table 2:** Patient-reported strength in relation to an objective strength assessment

Strength classification using percentage of normative dynamometer values		Reduced	Good	All	Sensitivity	Specificity	PPV	NPV
		(JAMAR <80 %), n	(JAMAR ≥80 %), n	n	(95 % CI)	(95 % CI)	(95 % CI)	(95 % CI)
ARM strength	Reduced (SIS 1–3)	64	10	74				
SIS 1a	Good (SIS 4–5)	9	16	25	0.87	0.62	0.86	0.64
	All	73	26	99	(0.779-0.942)	(0.410-0.800)	(0.766-0.933)	(0.425-0.820)
HAND strength	Reduced (SIS 1–3)	63	6	69				
SIS 1b	Good (SIS 4–5)	10	20	30	0.86	0.77	0.91	0.67
	All	73	26	99	(0.763-0.932)	(0.564-0.910)	(0.820-0.967)	(0.472-0.827)

Abbreviations: *CI*, 95 % confidence interval; Dynamometer; JAMAR Hand Dynamometer; *NPV*, Negative Predicted Value; *PPV*, Positive Predicted Value; *SIS*, Stroke Impact Scale questions 1A and 1B

## Discussion

Ten days after stroke, the majority of the patients’ perceptions of arm and hand strength were confirmed with results of an objective hand strength measure (hand held dynamometer), whereas some patients rated their strength differently. The discrepancy between objective strength and self-perceived strength highlights the importance of including self-perceived functioning early after stroke, combined with objective strength assessments to increase knowledge of a patient's awareness or lack thereof of their impairment. It is of clinical importance to assess the patient’s level of awareness in order to better help the patient to a successful rehabilitation [13] and to achieve rehabilitation goals. The use of self-perceived measurement may facilitate communication with the patient in assessment and rehabilitation processes as is encouraged in person-centered care [2].

As mentioned, a discrepancy between self-assessment and objective strength measurements has been shown previously [3, 4] and might occur within any part of the rehabilitation process. One study [29] reported reduced self-perceived function and less use of the impaired arm and hand at three months post stroke, although the patients were considered as being fully recovered based on objective measures. This confirms the need of a combined measurement strategy that is sensitive to change and assesses a broad range of performance [29]. However, correlations between self-reported and objective strength measures increases if the two methods reflect similar aspects of function (cover the same construct) [30]. It could be argued that self-reported assessment covers several constructs, as the patient’s perception probably includes evaluation of the strength in upper extremity when using the arm/hand in an activity. Objective measured strength assesses the actual strength in the particular measurement situation (one aspect). Despite the different constructs, the discrepancy and correlations were smaller than expected and previously reported [3]. An explanation for the differences in the present study could be that the majority of the patients were still in hospital, resulting in reduced need or cause to use the upper extremity in the activities of daily life. Other possible explanations to the differences could be reduced sensibility or co-coordination.

In order to increase the understanding of the relation between the perceived and objectively measured strength, a cut-off at 80 % of normative strength values [21] was used for comparison to self-perceived strength. The cut off at 80 % was chosen with the assumption that approximately 20 % of strength could be reduced without affecting performance in everyday tasks [12]. A large proportion of patients rated their strength low and was also objectively assessed as having reduced strength. The cut-off could however not explain all variation in self-perceived strength, indicated by the level of correctly classified patient of 81–84 %. The low negative predicted values could be explained by patients being unaware of, or neglecting their reduced strength which is of clinical relevance in rehabilitation. High positive predicted values indicated that patients with normal upper extremity strength assessed strength more correctly, than those who assessed reduced strength. Together, the result suggests that a patient’s rating of arm and hand strength is not sufficient to give valid information on strength, but is of importance in order to increase a therapist’s knowledge of a patient’s awareness of his/her strength. A patient who is unaware of physical problems [13], has poorer outcomes after rehabilitation as “awareness should be considered as the first building block in the rehabilitation process” and is required for a motivated patient [13]. Awareness of function also may facilitate setting of realistic goals in rehabilitation. With most recovery of function taking place within the first few weeks [8, 9], the self-perceived strength should be evaluated in the acute phase, with the purpose of identifying patients unaware of their problems, in order to enhance early rehabilitation planning.

It is important to enable use of structured questions covering the patients’ perspective very early after a stroke, but the availability of self-report instruments is limited. The SIS has been shown to be a patient-centered outcome measure with good responsiveness during the later stages post-stroke and to be useful in patients undergoing rehabilitation [31]. The use of the SIS has not been validated prior to one month post-stroke [19]. However, in the present study two questions from the SIS were assumed to have ecological validity in the present setting. With these aspects in mind, it was decided to use two questions of upper extremity strength, which focus on the strength during the last week (strength domain 1, SIS 1a and 1b). Although the choice of using SIS early after stroke may be seen as a limitation, however, three domains in the SIS (including the strength domain) assessed one month post-stroke, were recently shown to best indicate a patient’s primary problems at three and twelve months post-stroke [32].

A slight modification of the standardized assessment position of hand strength with the dynamometer (JAMAR) [20] was used in the present study. Patients were allowed to rest their arms on the table because the patients had large variations in upper limb function and this position allowed us to also measure patients with very little strength. This position may have affected the result in comparison to non-offloaded arm weight and without the stabilization in the shoulder that the support from the table gives. Another strength measurement method that could have been used to assess arm strength, is manual muscle testing. As this method has demonstrated insufficient validity [33] and would have prolonged the assessment procedure [34], we decided that this was not suitable for patients early after stroke.

There are some more issues in this study that need to be discussed. First, a dichotomization of SIS was undertaken in order to categorize patient’s self-assessment. A different choice of cut-off levels might have yielded a different result. Second, using percentage of normative values of JAMAR [21] is constrained by the size and background of the reference group. This might have affected the results in this study. Third, there are confounders that could have an impact on the results and limit the generalization. Cognitive deficits and language difficulties could have affected the accuracy on self-reported assessments after stroke [35]. The majority of the patients in the present study had no severely reduced cognitive function, as assessed by COG4 (similar precision as commonly used Mini-Mental State Examination). Other statistical methods could have been used to take these confounders into account, but in this material the size of subgroups did not allow multifactorial analysis. It should also be remembered that 18 patients from the SALGOT-population were excluded prior to the analysis process mainly due to severe communication disorder or cognitive deficits (5^th^ exclusion criteria). Taking these limitations in consideration, the findings from this study could be generalized to patients in an acute stroke unit with early start of rehabilitation, who have impaired upper extremity function without major cognitive defects. Further research is required to verify the results in other samples of patients, including those with cognitive or speech problems.

## Conclusion

This study shows in patients without severe cognitive impairment that despite high correlation between measures, patients’ perceptions of strength do not always correspond to an objective strength assessment. A combination of both self-reported and objective strength assessments in the upper extremity is needed to increase focus on the patient’s perspective and in goal setting in the initial days post-stroke.

## Abbreviations

ARAT: Action Research Arm Test
CI: Confidence Interval
JAMAR: JAMAR Hand Dynamometer
NIHSS: National Institute of Health Stroke Scale
NPV: Negative predicted values
PPV: Positive predicted values
SALGOT: Stroke Arm Longitudinal Study at the University of Gothenburg
SIS: Stroke Impact Scale
